# Till Death Do Us Part: Stable Sponge-Bacteria Associations under Thermal and Food Shortage Stresses

**DOI:** 10.1371/journal.pone.0080307

**Published:** 2013-11-28

**Authors:** Lucía Pita, Patrick M. Erwin, Xavier Turon, Susanna López-Legentil

**Affiliations:** 1 Departament de Biologia Animal and Institute de Recerca de la Biodiversitat (IRBio), Universitat de Barcelona (UB), Barcelona, Spain; 2 Center for Marine Science, University of North Carolina Wilmington, Wilmington, North Carolina, United States of America; 3 Center for Advanced Studies of Blanes (CEAB-CSIC), Blanes (Girona), Spain; Pennsylvania State University, United States of America

## Abstract

Sporadic mass mortality events of Mediterranean sponges following periods of anomalously high temperatures or longer than usual stratification of the seawater column (i.e. low food availability) suggest that these animals are sensitive to environmental stresses. The Mediterranean sponges *Ircinia fasciculata* and *I. oros* harbor distinct, species-specific bacterial communities that are highly stable over time and space but little is known about how anomalous environmental conditions affect the structure of the resident bacterial communities. Here, we monitored the bacterial communities in *I. fasciculata* (largely affected by mass mortalities) and *I. oros* (overall unaffected) maintained in aquaria during 3 weeks under 4 treatments that mimicked realistic stress pressures: control conditions (13°C, unfiltered seawater), low food availability (13°C, 0.1 µm-filtered seawater), elevated temperatures (25°C, unfiltered seawater), and a combination of the 2 stressors (25°C, 0.1 µm-filtered seawater). Bacterial community structure was assessed using terminal restriction fragment length polymorphism (T-RFLP) analysis of 16S rRNA gene sequences and transmission electron microscopy (TEM). As *I. fasciculata* harbors cyanobacteria, we also measured chlorophyll *a* (chl *a*) levels in this species. Multivariate analysis revealed no significant differences in bacterial T-RFLP profiles among treatments for either host sponge species, indicating no effect of high temperatures and food shortage on symbiont community structure. In *I. fasciculata*, chl *a* content did not significantly differ among treatments although TEM micrographs revealed some cyanobacteria cells undergoing degradation when exposed to both elevated temperature and food shortage conditions. Arguably, longer-term treatments (months) could have eventually affected bacterial community structure. However, we evidenced no appreciable decay of the symbiotic community in response to medium-term (3 weeks) environmental anomalies purported to cause the recurrent sponge mortality episodes. Thus, changes in symbiont structure are not likely the proximate cause for these reported mortality events.

## Introduction

Summer in the Western Mediterranean Sea is getting warmer and longer. Over the past decades, the frequency of seawater temperature anomalies and the period length of stable seawater column (i.e., stratification) have increased [1]–[3]. At the same time and coinciding with years of record temperatures (1–2°C above the mean summer temperature) or prolonged seawater stratification in late summer, mass mortality events were observed for several filter-feeding invertebrates, mainly sponges and cnidarians [3]–[5]. A typical summer season in the Mediterranean Sea is characterized by high temperatures (>18°C) that stratify the seawater column and prevent the upwelling of cooler nutrient-rich water, resulting in nutrient depletion, low turbidity and high irradiance in shallow waters (<20 m) [2]. Consequently, summer is a energetically-challenging season for filter-feeding invertebrates in the Mediterranean Sea [6], [7] and together with high temperatures or prolonged stratification, the additional physiological stress that occurs during this season may facilitate the observed episodes of mass mortality [2].

Marine sponges harbor diverse and host-specific bacterial communities [8], [9] suggesting that the ecology and survival of both the sponge and its bacterial associates are tightly connected; e.g. via nutrient translocation [10], [11]. However, despite the potential importance of sponge-bacteria interactions, to date few studies have experimentally assessed the response and stability of these associations under environmental conditions chosen to mimic realistic stress pressures. Most notably, manipulative experiments with the Great Barrier Reef sponge *Rhopaloeides odorabile* showed that the bacterial community associated with this sponge shifted in response to elevated temperatures, high nutrients and pollutants, concomitant with declines in host sponge health [12]–[15]. In temperate regions, sponge-derived bacterial communities changed when exposed to elevated temperatures [16] but remained stable under starvation conditions [17]. Further studies are needed to investigate the effect of extreme yet realistic environmental conditions on sponge-associated bacterial communities and assess their overall resilience amidst a changing climate.

Sponges in the genus *Ircinia* are ubiquitous in the Western Mediterranean rocky bottoms and harbor a species-specific bacterial community [18] that seems to be adapted to the seasonality of the water column [19]. Recently, *Ircinia* spp. have suffered dramatic episodes of mass mortality linked to extreme summer temperatures [20], [21] and the proliferation of an opportunistic *Vibrio*-like bacterium [21], [22]. The factors triggering the proliferation of *Vibrio*-like bacteria in sponge hosts remain unclear, but may be preceded by the disruption of the normal sponge microflora caused by abnormally high seawater temperatures lasting 3 weeks [20]. Cebrián *et al*. [20] observed significant reduction in photosynthetic efficiency in *I. fasciculata* individuals maintained in aquaria at elevated temperatures (27°C for 48 h). Based on these results, the authors suggested that cyanobacteria-harboring sponges such as *I. fasciculata* may be more susceptible to mass mortality events than other sponge species lacking photosymbionts.

In this study, we hypothesized that a high temperature treatment combined with low food availability mimicking an especially hot summer season in the Mediterranean Sea would be accompanied by a shift in the bacterial communities associated with Mediterranean sponges. Based on past studies [20], we expected that sponges harboring photosymbionts would be more susceptible to these shifts than those without them. To test these hypotheses, we performed a series of controlled aquaria experiments for the sympatric sponges *I. fasciculata* (which harbors cyanobacteria and has suffered mass mortality events) and *I. oros* (which does not harbor cyanobacteria, and has remained overall unaffected by mass mortality events). We tested the effect of high seawater temperature (25°C), food shortage (0.1 µm-filtered seawater) and the combination of both treatments on sponge-associated bacterial communities. Bacterial symbiont communities were monitored using terminal restriction fragment length polymorphism (T-RFLP) of 16S rRNA gene sequences and transmission electron microscopy (TEM) analyses. We also measured the concentration of chlorophyll *a* (chl *a*) in *I. fasciculata* samples as a proxy for photosymbiont abundance/activity in these hosts.

## Materials and Methods

### Specimen collection

40 individuals of the sponge *Ircinia oros* (Schmidt, 1864) and 40 of *I. fasciculata* (Pallas, 1766) were collected from shallow (<20 m) rocky reefs in the north-western Mediterranean Sea (Tossa de Mar, 41°43′13.62″ N, 2°56′26.90″ E) during January 2011 (*I. oros*) and February 2011 (*I. fasciculata*). Collection during winter months was favored for our experiments because temperatures are more stable during this period [19]. Within 2 h, the sponges were transported in insulated coolers from Tossa de Mar to the Experimental Aquaria Zone (ZAE) located at the Institute of Marine Science (ICM-CSIC) in Barcelona (Spain). *Ircinia* spp. are not endangered or protected by any law and all sampling was conducted outside protected areas following current Spanish regulations (no specific permits were required).

### Experimental design

Two experiment sets (one for each sponge species) were conducted in consecutive months, immediately after specimen collection. For each experiment, 40 specimens were placed in separated 2 L aquaria in a flow-through system with direct intake of seawater and an independent supply to each aquarium for a total of 4 weeks. The aquaria were subjected to circadian cycles of 12 h light/12 h dark using artificial light sources. The first week, sponges were maintained at natural (ambient) conditions as an acclimation period. During the following 3 weeks, 4 different treatments were set up (n = 10 individuals per treatment): non-filtered seawater and environmental temperature (control), 0.1 µm-filtered seawater and environmental temperature (FE), non-filtered seawater and hot temperature 25°C (NH), 0.1 µm-filtered seawater and hot temperature (FH). The environmental seawater temperature at the time of the experiments was 13°C. For the heat treatment, the temperature was progressively increased (ca. 1.5°C·day^−1^) during 7 days until reaching 25°C and then maintained at 25°C for the final 2 wk of the experiment. The health status of the sponges was monitored every 2 days by visual inspection for tissue necrosis. Water flux was also controlled every 2 days and readjusted if necessary to obtain a final flux rate through the aquaria of 0.8 L·min^−1^. Filters were replaced weekly to avoid flux reduction due to particle accumulation.

### Experimental sampling

Temperature (°C) and light intensity levels (lx  =  lumen·m^−2^) were recorded hourly with Hobo Pendant Temperature/Light Data Loggers (UA-002-64; Onset Computer Corporation). To check for filter efficiency and natural bacterial concentrations in the seawater, 3 samples of water per treatment were collected weekly, before filter replacement. Bacterial concentration was estimated by flow cytometry, based on the method described in Gasol & Del Giorgio [23]. In short, samples were fixed with 1% paraformaldehyde + 0.05% glutaraldehyde in a phosphate-buffered saline (PBS) solution, incubated in the dark for 10 min, deep frozen in liquid nitrogen and stored at −80°C. For analysis, samples were unfrozen, stained with Syto13 (Molecular Probes) at 5 µM (diluted in dymethil sulfoxyde, DMSO), incubated for 15 min in the dark and run through a GALLIOS flow cytometer with a laser emitting at 480 nm. Bacteria were detected according to a dot plot of side scatter (SSC, related with cell size) *versus* fluorescent signature (FL1). The number of events (potential bacterial cells) detected by the cytometer was then converted into bacterial cell density (cells·mL^−1^) by comparing with the events recorded by the machine after injecting a known volume of a solution of 10^6^ Syto13-stained beads·mL^−1^. For each sponge species, the non-parametric Mann-Whitney's U test was used to compare the bacterial cell density in seawater from non-filtered treatments *versus* filtered treatments. Statistical analyses were performed in RStudio [24]. All cytometry analyses were conducted at the Cytometry Unit of the Scientific and Technological Services of the University of Barcelona.

From all the sponge samples, we randomly selected 3 individuals per treatment that remained healthy throughout the experiment for further analysis (n = 24 per species). Overall, specimens of *I. fasciculata* and *I. oros* remained healthy in all experimental treatments with no tissue necrosis or appreciable biomass loss, except for 1 individual of *I. fasciculata* that died during the acclimation period, and 5 individuals of *I. oros* that died during the first week of experiment (1 from the FE treatment, 3 from the NH, and 1 from the FH). These specimens were not considered in our analysis for several reasons: (i) death was likely due to manipulation rather than to the tested conditions because they all died early during the experiments; (ii) by the end of the experiment, the sponges had been dead for at least two weeks (iii) there were insufficient replicates for robust statistical analysis.

### DNA extraction

After the acclimation period (end of week 1) and at the end of the experiments (end of week 4), a tissue sample (ca. 2 mm^3^) of each selected specimen containing both ectosome and choanosome was preserved in 100% ethanol and stored at −20°C. To characterize the bacterial community in the seawater, 500 mL of water per treatment were filtered through a 0.2 µm filter (Millipore), preserved in 100% ethanol and stored at −20°C. DNA was extracted using the DNeasy Blood & Tissue kit (Qiagen®). Dilutions (1∶10) of DNA extracts were used as templates in subsequent PCR amplifications for T-RFLP analysis.

### T-RFLP analysis

PCR amplification of 16S rRNA gene sequences was conducted using the universal bacterial forward primer Eco8F [25], tagged with a 5′-6-carboxyfluorescein (6-FAM) label, and reverse primer 1509R [26]. PCR was performed as follows: one initial denaturation step for 5 min at 94°C; 35 cycles of 1 min at 94°C, 0.5 min at 50°C, 1.5 min at 72°C; and one final elongation step for 5 min at 72°C. Total PCR volume (50 µL) included 10 µM of each primer, 10 nM of each dNTP, 1x Reaction Buffer (Ecogen), 2.5 mM MgCl_2_ and 5 units of BioTaq™ DNA polymerase (Ecogen). Products from triplicate PCR reactions were pooled and purified from electrophoresis gels using the Qiaquick Gel Extraction kit (Qiagen®), then quantified using the Qubit™ fluorometer and Quant-iT™ dsDNA Assay kit (Invitrogen™) according to manufactures' instructions. Separate enzymatic digestions with *Hae*III and *Msp*I were processed as described elsewhere [27], then analyzed in an automated ABI 3730 Genetic Analyzer (Applied Biosystems) at the Genomics Unit of the Scientific and Technological Services of the University of Barcelona. The lengths of each terminal-restriction fragment (T-RF) were determined against a size standard (600-LIZ) using the PeakScanner™ software (Applied Biosystems). T-RFs smaller than 50 bp or larger than 600 bp were discarded because they were beyond the resolution of the size standard. Background noise was defined by a peak intensity below 50 fluorescence units and by filtering in T-REX [28] using a cut-off value of 2 standard deviations [29]. ‘True’ T-RFs were aligned in T-REX using a clustering threshold of 1 bp and relative T-RF abundance matrices were constructed.

### T-RFLP statistical analyses

Samples from each experimental set were analyzed separately to investigate whether the observed response to each treatment depended on sponge species (*I. fasciculata* and *I. oros*). All analyses were based on Bray-Curtis distances calculated from relative abundance matrices, following square root transformation. For each restriction enzyme, non-metric multi-dimensional scaling (nMDS) plots were constructed to visually compare the bacterial communities. Permutational multivariate analyses of variance (PERMANOVA) [30], [31] were used to test the effects of source (sponge or seawater) and treatment (control, FE, NH, FH) on bacterial communities. In addition, sponge samples collected after the acclimation period were compared to verify that the specimens harbored similar bacterial communities before experimental treatments were applied. Calculations were performed in PRIMER v6 [32], [33] and PERMANOVA+ (Plymouth Marine Laboratory, UK). The empirical T-RFs obtained in this study were compared with the available database of *in silico Hae*III and *Msp*I digestions of 16S rRNA gene sequences derived from the same host sponges in a previous study [19] using the phylogenetic assignment tool PAT [34].

### Transmission electron microscopy (TEM)

At the end of the experiments, a piece of tissue (ca. 2 mm^3^) from one sponge in each treatment was collected and fixed in a solution of 2.5% glutaraldehyde and 2% paraformaldehyde buffered with filtered seawater and incubated overnight at 4°C. Following fixation, each piece was rinsed at least three times with filtered seawater and stored at 4°C until processed as described previously [35]. TEM observations were made at the Microscopy Unit of the Scientific and Technical Services of the University of Barcelona on a JEOL JEM-1010 (Tokyo, Japan) coupled with a Bioscan 972 camera (Gatan, Germany).

### Chlorophyll *a* (chl *a*) concentrations

For chl *a* quantification in *I. fasciculata*, a piece of ectosome was sampled from 5 sponges per treatment at the end of the experiments (n = 20) and processed them using previously described methods [18]. *I. oros* was excluded from this analysis because this species lacks photosymbionts [18]. One-way ANOVA was performed to test the effect of the factor “treatment” (4 levels; control, FE, NH, FH) on chl *a* concentrations in *I. fasciculata*. The assumptions of the ANOVA were checked by Cramer-von Mises' normality test and Levene's homoscedasticity test. Statistical analyses were performed in RStudio [22].

## Results

### Aquaria conditions

Artificial light intensity in the aquaria with *I. fasciculata* samples was 546.7±25.0 lx (mean ± standard error) and in the aquaria with *I. oros* 644.1±8.9 lx. Both light intensity values were in the range of values detected in their natural habitat during winter [19]. Environmental water temperature was 13.42±0.01°C and 13.54±0.18°C (mean ± standard error) for the experiment with *I. fasciculata* and with *I. oros*, respectively. For hot temperature treatments, temperature was increased at a rate of 1.49°C·day^−1^ for the aquaria with *I. fasciculata* samples and 1.57°C·day^−1^ for *I. oros* samples during one week, until reaching a final temperature of 25.41±0.01°C and 25.23±0.05°C (mean ± standard error) for the experiment with *I. fasciculata* and with *I. oros*, respectively. The average densities (mean ± standard error) of bacterial cells found in seawater samples from the filtered treatments were (2.4±0.3)·10^4^ cells·mL^−1^ in *I. fasciculata* aquaria, and (2.3±0.2)·10^4^ cells·mL^−1^ in *I. oros*, while in the unfiltered treatments contained (7.4±1.0)·10^4^ cells·mL^−1^ and (6.8±0.5)·10^4^ cells·mL^−1^ in aquaria with *I. fasciculata* and *I. oros*, respectively. In spite of the filtering system, bacterial abundance was only cut by ca. one third. This may relate with decaying filter efficiency with time, in spite of weekly filter changes. Still, the bacterial cell density in seawater samples from non-filtered treatments was statistically higher than in filtered treatments (Mann-Whitney's U, *P*<0.001) for both *I. fasciculata* and *I. oros* experiments. A one-third reduction in bacterial density is likely a realistic proxy for food shortage conditions in nature.

### T-RFLP analysis

**Figure 1:**
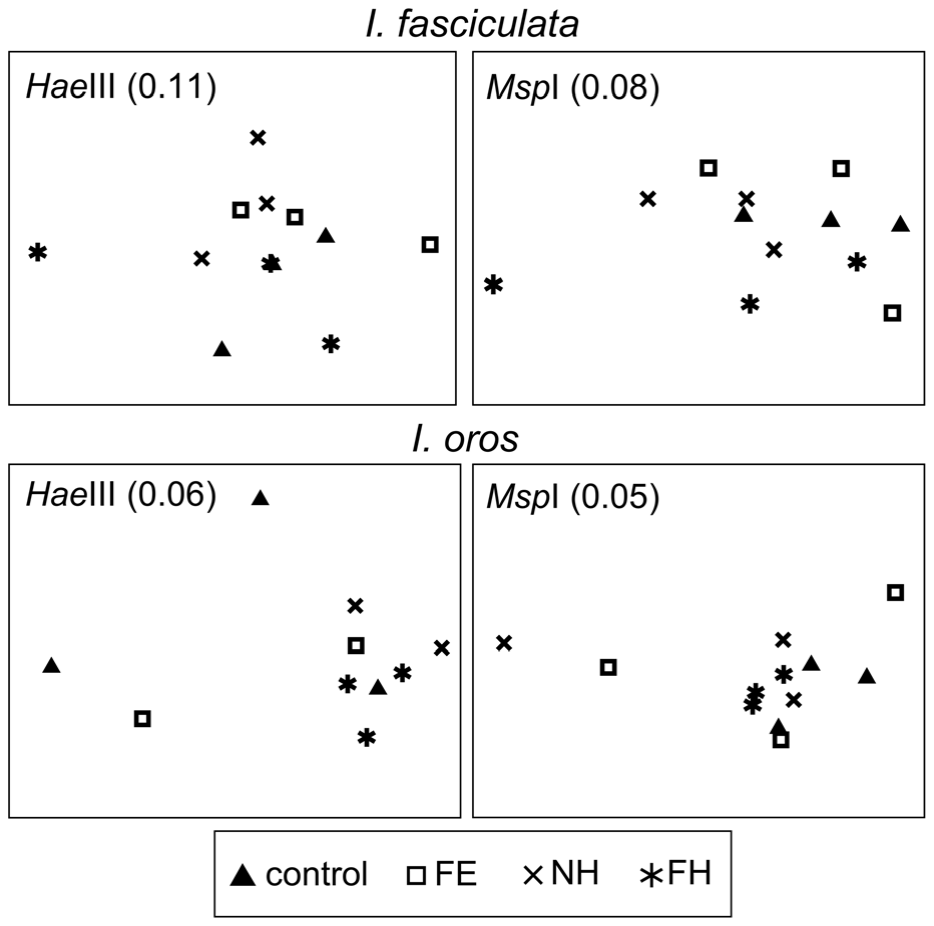
Non-metric multidimensional scaling (nMDS) of sponge-derived bacterial communities at the end of the experiment. Ordination in nMDS plots is based on Bray-Curtis distances between T-RFLP profiles from *Hae*III (left) and *Msp*I (right) digestions of samples of *I. fasciculata* and *I. oros* experiments. Stress values are shown in parenthesis, with values below 0.15 indicating good correlation of similarity matrix distances and ordination in the two-dimension plot. Points are coded by treatment: control (13°C, unfiltered seawater), FE (13°C, filtered seawater), NH (25°C, non-filtered seawater), FH (25°C, filtered seawater).

A total of 143 unique T-RFs were detected with *Hae*III restriction enzyme (101 in *I. fasciculata*, 97 in *I. oros* and 59 in seawater) and 167 with *Msp*I enzyme (117 in *I. fasciculata*, 110 in *I. oros* and 79 in seawater). PERMANOVA analysis of Bray-Curtis similarity matrices from each experiment reported a significant effect of source (sponge *vs* seawater) on the structure of bacterial communities (Table 1). No significant differences in bacterial community structure were detected among samples of the same sponge species after the acclimation week (*P*>0.225, for both enzymes). Likewise, there was not a significant effect of treatment on the bacterial communities of *I. fasciculata* and *I. oros* after 3 weeks (Table 1). As the experiment was terminated after 3 weeks, there is no data beyond the duration of the experiments. The lack of structure observed with the nMDS plots further confirmed the similarity of these bacterial communities within host species, despite the different treatments applied (Fig. 1). PAT analysis reported that 58.7% (*Hae*III) and 71.6% (*Msp*I) of the unique T-RFs obtained in this study for both *I. fasciculata* and *I. oros* matched T-RFs from *in silico* digestions of 16S rRNA sequences from environmental samples of these two species [18].

**Table 1:** Statistical analysis of T-RFLP profiles to test for an effect of source (seawater *vs* sponge) and treatment on the structure of *Ircinia*-associated bacterial communities.

	*I. fasciculata*		*I. oros*	
	*Hae*III	*Msp*I	*Hae*III	*Msp*I
**Source** (seawater *vs* sponge)	**0.001**	**0.001**	**0.001**	**0.002**
**Treatment** (control, FE, NH, FH)	0.317	0.328	0.267	0.066

Numbers denote *P*-values from PERMANOVA test after 999 permutations. Significant values at *α* = 0.01 are in bold.

Treatments: Control (13°C, unfiltered seawater), FE (13°C, filtered seawater), NH (25°C, non-filtered seawater), FH (25°C, filtered seawater).

### Transmission electron microscopy

**Figure 2:**
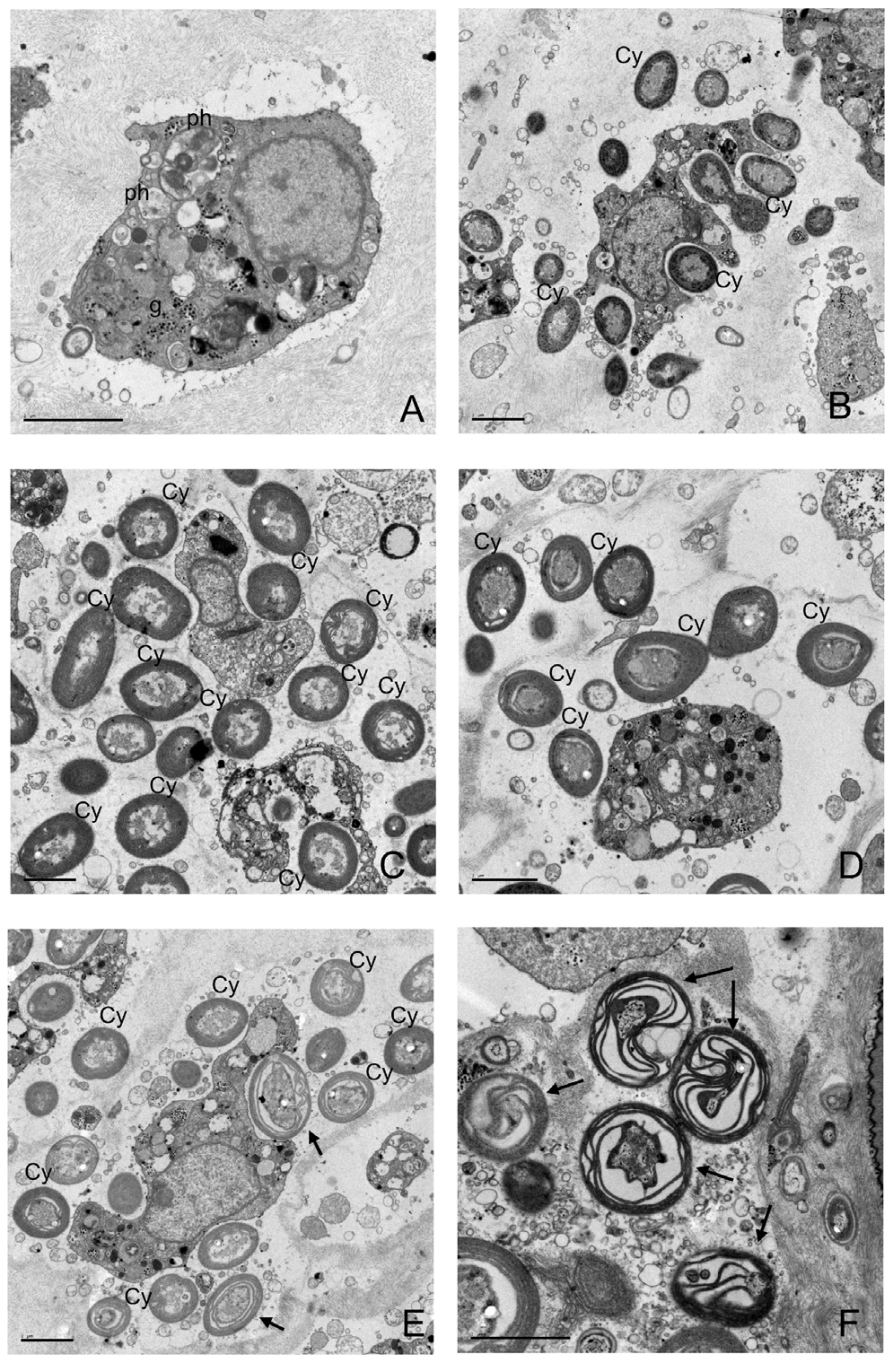
Electron micrographs of *I. fasciculata* bacteria at the end of the experiment. A) Sponge cell in sample from control treatment, containing several phagosomes (ph) and glycogen granules (g). Sponge cells surrounded by multiple *Cyanobacteria* (Cy) and heterotrophic bacteria in the mesohyl of sponges from control treatment (B), NH (25°C, non-filtered seawater) treatment (C) and FE (13°C, filtered seawater) treatment (D). Micrographs of a sponge from FH (25°C, filtered seawater) treatment (E, F) showed healthy *Cyanobacteria* (Cy) and *Cyanobacteria* under different stages of degradation (arrows) within the mesohyl. Scale bars represent 2 µm.

**Figure 3:**
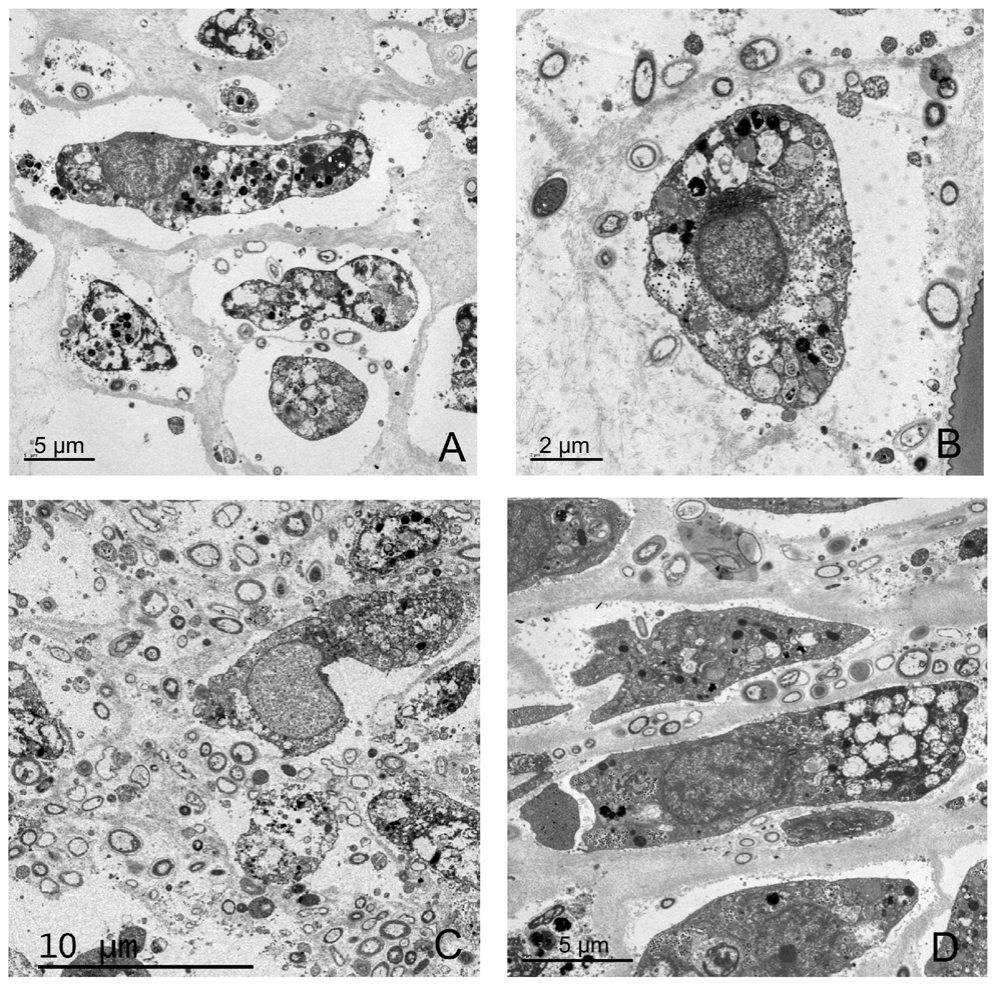
Electron micrographs of *I. oros* bacteria at the end of the experiment. Sponge cells surrounded by numerous bacteria cells of different morphotypes. Samples from control treatment (A); 25°C and non-filtered seawater treatment (B); 13°C and filtered seawater treatment (C); and 25°C and filtered seawater treatment (D). Sponge and bacteria cells for all treatments showed no sign of degradation.

Micrographs of *I. fasciculata* samples from the control treatment showed typical sponge cells with numerous phagosomes and granules of glycogen (Fig. 2a). The same sponge cells were observed in all the other treatments. The cyanobacterium *Candidatus* ‘Synechococcus spongiarum’ dominated the ectosomal tissue of *I. fasciculata* (Fig. 2b–e). In the micrographs from the hot temperature (25°C) and filtered seawater treatment (FH), besides healthy cyanobacterial cells, we also observed many cells undergoing degradation (Fig. 2e–f). Electron micrographs from *I. oros* samples (Fig. 3a–d) showed abundant vacuolated sponge cells surrounded by diverse bacterial morphotypes. No differences in sponge or bacterial cell abundance or morphology were detected for any of the treatments. As expected, no cyanobacterial cells were observed either in this sponge species.

### Chlorophyll *a* concentration

**Figure 4:**
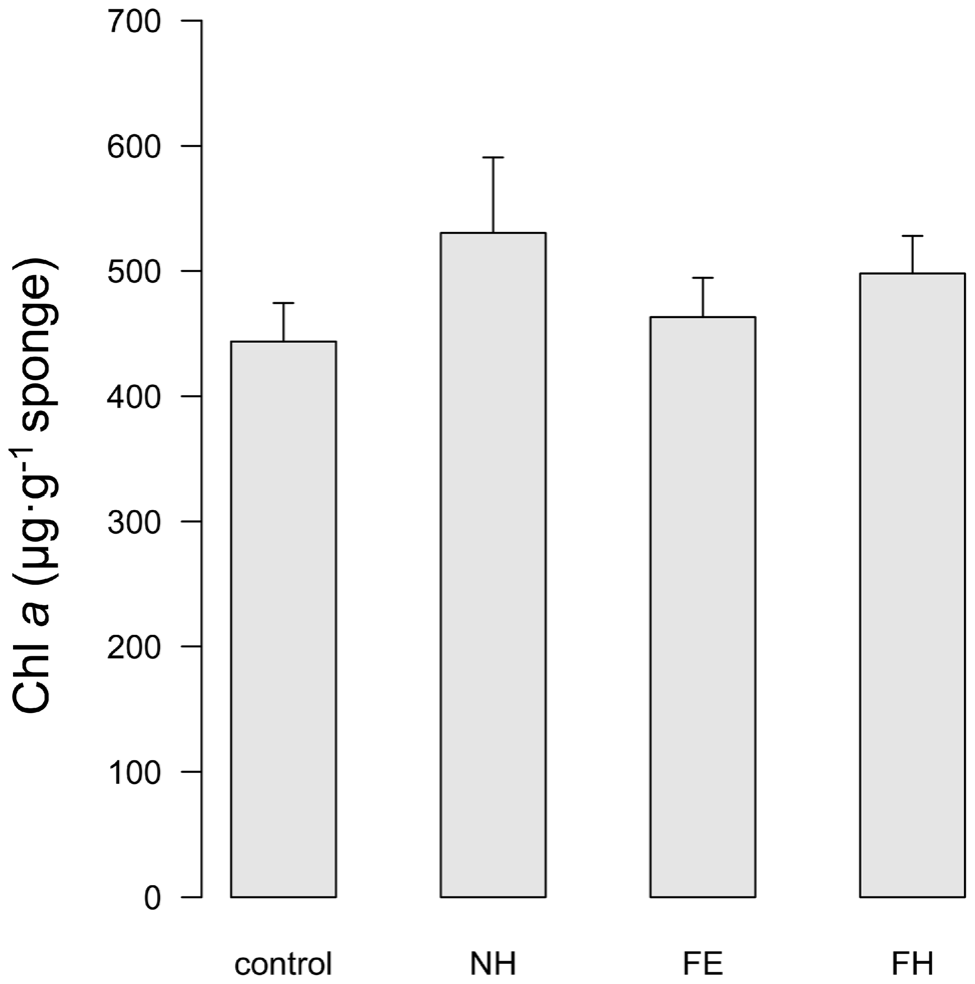
Chlorophyll *a* concentration in *I. fasciculata* for each treatment at the end of the experiment. Control: 13°C and non-filtered seawater; NH: 25°C and non-filtered seawater; FE: 13°C and filtered seawater; FH: 25°C and filtered seawater. Error bars denote standard error.

Chl *a* levels in *I. fasciculata* at the end of the experiment (3 weeks after acclimation) and for each treatment are depicted in Fig. 4. The ANOVA test revealed no significant differences in chl *a* concentration among treatments (*P* = 0.4636). The values found here (483.8±20.0 µg·g^−1^ sponge, mean ± standard error) exceeded those observed for this species in the field, where the average concentration reported was 248.1±27.8 µg·g^−1^ sponge [19].

## Discussion

The bacterial communities associated with the Mediterranean sponges *I. fasciculata* and *I. oros* were stable under thermal and food shortage stresses for a period lasting 3 weeks. Comparison of T-RFLP profiles and electron microscopy for each species showed no significant differences among the 4 treatments tested that combined high seawater temperatures (25°C) and low food availability (one-third reduction of the natural bacterial abundance) during three weeks after acclimation. The only noticeable difference consisted of TEM observations of several degraded cyanobacterial cells of *S. spongiarum*, along with healthy looking ones, when *I. fasciculata* specimens were exposed to both thermal and food shortage stresses. However, the presence of degraded cells was not accompanied by a significant decrease in chl *a* concentrations. In fact, chl *a* content was higher in our aquaria samples and for all treatments than what has been observed in the field [19]. This increase in chl *a* concentration may be due to a higher density of cyanobacterial cells in the sponge or enhanced photosynthetic activity to compensate for lower ambient irradiance conditions or a poorer diet. Overall, our results indicate that the seawater conditions that characterize anomalously warm summer seasons in the Mediterranean Sea do not affect sponge-associated bacterial communities. Moreover, we did not observe any clear evidence supporting the hypothesis that sponges harboring cyanobacterial symbionts were more vulnerable to the assayed conditions than sponges without them. Other species-specific factors such as habitat-preference or growth dynamics [34], alone or in combination, may contribute to the sporadic mass mortality events observed for *I. fasciculata* but not for *I. oros* in the Mediterranean Sea.

One specimen of *I. fasciculata* and 5 of *I. oros* died during the experiments and were excluded from T-RFLP analysis. Necrosis in *I. fasciculata* occurred during the acclimation period and thus was unrelated with the tested treatments. Individual plasticity in resilience to collection and transport or health status at the moment of sampling may have affected the survival of that specimen when moved into aquaria. For *I. oros* sponges, death occurred early during the second week, before the targeted elevated temperature was reached, and sporadically among treatments. Previous studies assaying similar thermal stressors have reported host tissue necrosis and symbiotic cyanobacterial loss in all specimens at elevated seawater temperatures after only 3 to 4 days of treatment [13], [14]. While we cannot be certain of the reason behind the death of these few sponges (i.e. tested treatments or different response to maintenance in aquaria), none of our treatments resulted in mass mortality and the remaining specimens looked healthy through the 3-week experiment.

We cannot disregard that longer-term experiments (months) could result in a significant effect of treatment on bacterial community structure. Stratification of the water column along the Mediterranean coast lasts more than three weeks. Nevertheless, the persistence reported in this study is still remarkable. The high temperature tested here (25°C) represents 3°C more than the summer mean temperature in the study area [19], matched the maximum temperature detected during anomalous summer seasons in years when mass mortality events occurred [20], and represents an increase of >11°C from ambient conditions at the time of collection. In addition, the time frame of our experiments (3 weeks after acclimation) matched the duration of peaks of temperature in abnormally warm summers [20].

Our results are also in agreement with other studies indicating that sponge-bacteria associations are very stable and able to resist non-lethal stressful conditions. In the Mediterranean sponge *Aplysina aerophoba*, neither food shortage nor antibiotic exposure promoted the consumption of symbionts by the host and the structure of the bacterial community remained unchanged for up to 11 days [17]. In the tropical sponge *Rhopaloeides odorabile*, the bacterial community shifted only when sponge tissue necrosis occurred, after exposure to temperatures 2 to 4°C above the mean temperature in the study area [13], [14]. Interestingly, Fan et al. [35] observed that the expression of genes potentially essential for the symbiotic relationship (e.g. proteins involved in cell-cell signaling that could mediate recognition of symbiont by host) was maintained in partially necrotic sponges although at a lower rate than in healthy ones.

Despite the overall stability of sponge-associated bacteria, cells of dominant cyanobacterium *S. spongiarum* were observed undergoing degradation in *I. fasciculata* sponges exposed to high temperature and food shortage stresses (FH). While not all *S. spongiarum* cells were degrading and chl *a* content did not differ among treatments, the observation of this phenomenon only in the most stressful treatment suggests higher sensitivity of cyanobacteria to these conditions. Previous studies indicated that cyanobacteria-harboring sponges were more vulnerable to elevated temperatures due to photo-oxidative stress (i.e., rising levels of harmful oxygen compounds) derived from temperature-enhanced photosynthesis [20]. However, the stability of the symbiotic community and cyanobacterial chl *a* content across treatments observed in this study suggest that the overall photosynthetic activity was not impaired by the degradation of some cyanobacterial cells and that the sponge holobiont is able to resist these conditions for 3 weeks.

The persistence of bacterial symbiont communities despite thermal stress and food shortage conditions lasting 3 weeks is in opposition to one of the predictions of the coral probiotic hypothesis [36]. According to this hypothesis, the microbial symbionts associated with corals would rapidly shift in response to changing environmental conditions (in days to weeks), thereby conferring an adaptive response to the host. In sponges, it does not seem that rapid changes in bacterial community structures would provide stress tolerance to the host [13]. Instead, we speculate that, similar to what has been proposed for the human gut microbiome [37], a persistent symbiotic community in sponges results in constitutive benefits, such as preventing the unexpected proliferation of one or a few bacterial strains within the symbiotic community that yield holobiont death. The empirical demonstration of interactions within the bacterial community and between the bacteria and host that maintain the stability of the symbiotic community under environmental stresses remains a challenge for sponge microbiology.

In conclusion, our experiments for the sympatric sponges *I. fasciculata* and *I. oros* maintained in aquaria mimicking an especially hot summer in the Mediterranean Sea revealed high persistence of sponge-associated bacterial communities. These findings support trends observed in the field showing high symbiont stability across spatial and temporal scales [19], [38], [39] and also suggest that the disruption of the symbiotic community in response to abnormal thermal and food shortage conditions for a period up to three weeks may not be the primary cause of the sporadic mass mortality events observed for some *Ircinia* species.
